# WNT7B in fibroblastic foci of idiopathic pulmonary fibrosis

**DOI:** 10.1186/1465-9921-13-62

**Published:** 2012-07-28

**Authors:** Travis Meuten, Ariel Hickey, Katherine Franklin, Brian Grossi, Jeremy Tobias, Donna R Newman, Samuel H Jennings, Maria Correa, Philip L Sannes

**Affiliations:** 1 Departments of Molecular Biomedical Sciences, College of Veterinary Medicine, North Carolina State University, 1060 William Moore Dr, Raleigh, NC 27607, USA; 2 Population Health and Pathology, enter for Comparative Medicine and Translational Research, College of Veterinary Medicine, North Carolina State University, Raleigh, NC, USA

**Keywords:** Myofibroblasts, Alveolar epithelium, Interstitial lung disease

## Abstract

**Background:**

Idiopathic pulmonary fibrosis (IPF) is a devastating interstitial pneumonia causing a loss of respiratory surface area due to a proliferative fibrotic response involving hyperplastic, hypertrophic, and metaplastic epithelium, cystic honeycomb change, septal expansion, and variable inflammation. Wnt (wingless) signaling glycoproteins are known to be involved in lung development and tissue repair, and are up-regulated in patients with IPF. Based on previous qRT-PCR data showing increased Wnt7B in lungs of IPF patients, a systematic, quantitative examination of its tissue site distribution was undertaken.

**Methods:**

Tissue samples from the Lung Tissue Research Consortium (LTRC) of 39 patients diagnosed with mild to severe IPF/usual interstitial pneumonia (UIP) and 19 normal patients were examined for the immunolocalization of Wnt7B.

**Results:**

In normal lung, moderate Wnt7B reactivity was confined to airway epithelium, smooth muscle of airways and vasculature, and macrophages. IPF lung showed strong Wnt7B reactivity in fibroblastic foci, dysplastic airway and alveolar epithelium, and in highly discrete subepithelial, basement membrane-associated regions. All reactive sites were sized and counted relative to specific microscopic regions. Those in the subepithelial sites were found in significantly greater numbers and larger relative area compared with the others. No reactive sites were present in normal patient controls.

**Conclusions:**

The results demonstrate Wnt7B to be expressed at high concentrations in regions of active hyperplasia, metaplasia, and fibrotic change in IPF patients. In this context and its previously established biologic activities, Wnt7B would be expected to be of potential importance in the pathogenesis of IPF.

## Introduction

Idiopathic pulmonary fibrosis/usual interstitial pneumonia (IPF/UIP) is a debilitating disease characterized by a loss of normal respiratory architecture and replacement with a heterogeneous population of myofibroblast-like cells and excess of fibrous connective tissue restricted to the lung [1,2]. IPF arises from inflammation in the alveolar-capillary wall resulting in alveolar type I cell (AT1) loss and AT2 cell hyperplasia and subepithelial/interstitial fibrogenesis [3,4]. It has been suggested this represents an attempt to repair the pulmonary barrier following an injury to the respiratory surface [5]. The hallmark lesions are fibroblastic foci, signifying active disease, with a patchy mix of older fibrosis starting from the subpleural surface and along interlobular septa. Early lesions frequently appear highly cellular, with subepithelial fibroblastic foci adjacent to normal pulmonary architecture [6,7]. In IPF the lesions lead to end-stage fibrosis with minimal remaining pulmonary structure [6-8]. The proposed pathogenesis centers on dysregulation of epithelial repair in the form of hyperplastic and metaplastic AT2 cells interrelated with fibroproliferative lesions and aberrant epithelial differentiation, including epithelial to mesenchymal transition (EMT) [8,9].

The signaling pathways involved are only partially understood. It is known that there is increased expression of TGF-β and α-smooth muscle actin (α-SMA) in progressive lesions of IPF reflecting the transition of fibroblasts to myofibroblasts with proliferation and collagen maintenance modulated by wingless (Wnt) glycoproteins [10-12]. Mouse models indicate cooperation of TGF-β and Wnt signaling pathways in development, differentiation, and EMT [13]. Altered expression of Wnt ligands and one of their downstream targets, β-catenin, is evident in early IPF and bleomycin models of pulmonary fibrosis [14-16]. This can be explained in light of up-regulated TGF-β by its induction of LEF-1, which is a component of the canonical Wnt signaling pathway [13]. Chilosi, et al., found highly concentrated sites of β-catenin in myofibroblast-populated regions adjacent to airways [14]. This correlated with elevated mRNA expression and immunohistochemical reactivity of Wnts 1 & 3a in adjacent pulmonary epithelium in IPF patients [17]. Recent evidence shows that Wnt3a can activate β-catenin-mediated signaling and induce EMT in lung epithelial cells [18]. The collective views suggest that canonical Wnt signaling (β-catenin mediated) is up-regulated in fibrogenic conditions and may be causally involved in IPF [16-19].

Wnt glycoproteins have been found to be expressed at low levels in the normal lung and may be associated with epithelial turnover [17,20]. For example, Wnt7B mRNA expression in isolated normal AT2 cells was evident in low levels [17,21]. Wnt7b over-expression is thought to be a contributing factor to fibrogenesis in the murine kidney [22] and to be involved with procollagen production by lung fibroblasts [12]. Wnt7b was of special interest because of its role in mesenchymal proliferation and vascular development in the lung [23]. Based on supporting qRT-PCR data showing increased Wnt7B in lungs of IPF patients [17], a systematic, examination of its localization in a cohort of 39 IPF lungs was undertaken. Wnt7B expression was found in spindle cells and extracellular matrix of virtually all fibroblastic foci and in widely distributed, large numbers of discretely defined regions of the subepithelial basement membrane zone. These findings may provide useful clues relating to the pathogenesis of IPF and present novel, potential targets for detection and treatment.

## Materials & methods

### Immunostaining

Tissue blocks of formalin-fixed lung tissue samples were obtained from the Lung Tissue Research Consortium (LTRC). The samples had previously been placed into three groups (see Table1): group 1, with forced vital capacities (FVCs) >80% (normal, or no specified major or minor diagnosis, n = 19); group 2, with FVCs between 50-80% [major final clinical diagnosis as interstitial lung disease (ILD, n = 20) and minor final clinical diagnosis as usual interstitial pneumonia (UIP)/idiopathic pulmonary fibrosis (IPF)]; and group 3, with FVCs <50% (major final clinical diagnosis of ILD and minor final clinical diagnosis as UIP/IPF, n = 19). No other patient identifiers were provided, and their anonymity and confidentiality were preserved. The study was approved by the North Carolina State University Institutional Review Board. Blocks were further randomized and sections stained with hematoxylin and eosin (H&E) and picrosirius red (PSR) or Movat’s pentachrome stain for collagen. H&E sections were examined by a Board Certified Pathologist to independently confirm/reclassify initial clinical diagnoses.

**Table 1:** Patient group data summary and total Wnt7B area reactivity

**Patient ID**	**Area Total μm**^**2**^**/mm**^**2**^	**Path Diagnosis FVC > 80%**	**Patient ID**	**Area Total μm**^**2**^**/mm**^**2**^	**Path Diagnosis FVC > 50% < 80%**	**Patient ID**	**Area Total μm**^**2**^**/mm**^**2**^	**Path Diagnosis FVC < 50%**
34/1	0	Normal	22/29	0.22660864	Mild IF	53/13	0.308239253	Mild IF
50/8	0	Normal	52/59	0.46500093	Mild IF	40/11	0.318122907	Mild IF
60/9	0	Normal	9/55	0.702668072	IPF	51/7	0.421600843	IPF/UIP
29/25	0	Normal	46/51	0.792223807	IPF	32/28	0.543860737	IPF/UIP
27/26	0	Normal	3/50	0.853712645	Severe IF	11/12	0.609837285	IPF/UIP
43/16	0	Normal	39/43	0.898132637	IPF/UIP	2/38	0.689568308	IPF/UIP
33/23	0	Normal	30/17	0.984128952	IPF/UIP	38/35	0.870788258	IPF/UIP
13/39	0	Mild emphysema	58/15	1.02457832	Mild IF	19/47	1.07500215	Mild IF
47/44	0	Normal	54/34	1.124896765	IPF/UIP	23/31	1.246080924	Inf/Honeycombing
31/45	0	Normal	12/19	1.127274982	Severe IF	48/53	1.332033914	Mild IF
20/46	0	Normal	55/5	1.135880134	Mild IF	35/10	1.451763467	IPF/UIP
28/49	0	Mild emphysema	15/4	1.19162088	Mild IF	16/21	1.506947458	IPF/UIP
5/52	0	Septal thickening	42/32	1.257089128	IPF/UIP	49/20	1.597695503	Mild IF
1/57	0	Normal	45/18	1.470515762	IPF/UIP	4/41	1.698264266	IPF/UIP
7/60	0	Normal	36/33	1.5500031	IPF/UIP	10/22	2.0666708	Mild IF
57/36	0	Mild emphysema	24/42	1.667114445	IPF/UIP	25/40	2.214.290	IPF/UIP
14/3	0.077500155	A telectasis/thickening	37/30	2.258029207	IPF/UIP	21/37	2.188697845	Mild IF
8/27	0.116043013	Normal	18/2	2.294004588	DIFFUSEIF	56/24	2.279055942	IPF/UIP
41/14	0.41333416	Normal	44/56	2.679444431	IPF/UIP	17/48	2.605680887	IPF/UIP
			59/58	3.620364384	IPF/UIP			
avg	0.031940912		avg	1.366149591		avg	1.267217264	
stdev	0.097448895		stdev	0.808903868		stdev	0.71592836	

Legend: *IF* – interstitial fibrosis; *IPF* – idiopathic pulmonary fibrosis; *UIP* – usual interstitial pneumonia.

Sections were treated with citrate buffer for antigen retrieval and treated with a polyclonal goat anti-Wnt7B antibody [Santa Cruz Biotechnology, Inc. (sc26363, lot# I0205, Santa Cruz, CA)] at a 1:100 dilution overnight at 4°C, followed by peroxidase-labeled secondary antibodies (Dako LSAB+, Dako Laboratories, Carpinteria,CA) and Nova Red (Vector Laboratories, Burlingame, CA). The Wnt7B antibody recognizes both precursor and mature forms of human origin. Control samples substituted normal goat serum for the primary antibody, or were pre-treated with 5% testicular hyaluronidase (Sigma, Type 1-S, St. Louis, MO) for 30 minutes at 37°C to release Wnt7B bound to extracellular matrices [24], or treated with a competitive antibody-binding Wnt7B peptide to the primary antibody incubation. Sections were counterstained with methylene blue. Selected sequential and non-sequential serial sections were immunostained with anti-human smooth muscle actin.

### Analysis of Reactive Sites

The entirety of each section was systematically evaluated at 200X magnification through a calibrated ocular grid, and the size of each Wnt7B reactive site was measured. They were divided into three major size categories: <50 μm^2^, 50–100 μm^2^, and >100 μm^2^; while their histologic sites were separated into one of three regional designations: 1) epithelium and underlying fibroblasts with or without extracellular matrix (EF), 2) subepithelial fibroblasts and ECM (SE) without epithelial staining (included most fibroblastic foci), and 3) interstitial fibroblasts and ECM (I) that were not directly subjacent to an epithelial surface. The number of sites corresponding to the respective categories was recorded. The total surface area, as defined by the external border of each tissue section, was determined using ImageJ (National Institutes of Health, Bethesda, MD), and the percent of the total area of each reactive site category relative to the total section surface area was calculated (see Table1).

### Statistical analysis

Comparisons of the descriptive statistics of reactive sites were made using Analysis of Variance or Kruskal-Wallis non-parametric test for histologic locations in the same reactive site groups and within lung capacity category using SAS software (Cary, NC) or Minitab (Six Sigma, State College, PA). For example, for patients in FVC 50-80%, comparisons were made for reactive sites >100 (μm^2^) for EF, SE, and I locations. Statistical significance was set at an alpha value of ≤ 0.05.

## Results

**Figure 1:**
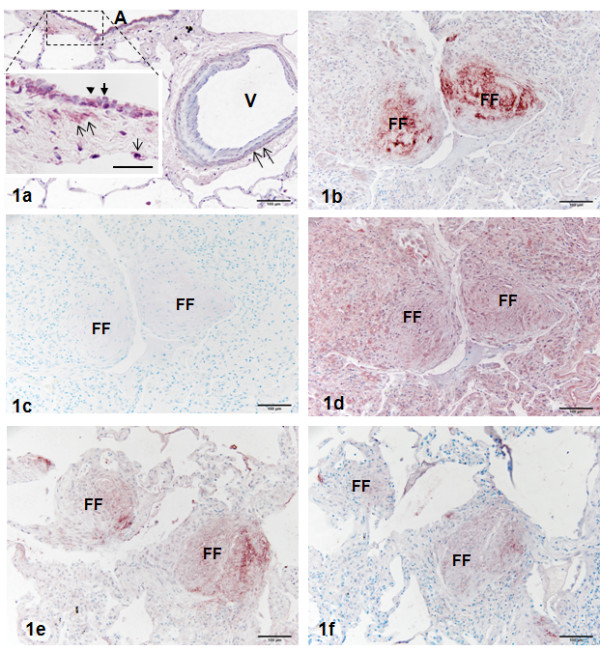
**Section of normal human lung treated for the immunohistochemical localization of Wnt7B.** Figure1a at low power, airway (A) epithelial cells and smooth muscle (double arrows) surrounding a nearby vessel (V) exhibit reactivity for Wnt7B. Inset of the airway shows Wnt7B reactivity in ciliated cells (solid arrowtip), AT2 cell (single arrow), and smooth muscle (double arrows). The airway insert also shows and non-reactive non-ciliated bronchiolar cell (solid arrowhead). Methylene blue counterstain. Bar = 100 μm; inset Bar = 30 μm. Figure1b, Section of UIP/IPF lung treated for the immunohistochemical localization of Wnt7B. Two large fibroblastic foci (FF) demonstrate strong extracellular reactivity. Bar = 100 μm. Figure1c, Serial section of UIP/IPF lung in Figure1b treated with non-immune serum instead of the Wnt7B-specific antibody. Fibroblastic foci (FF) lack selective staining for the antigen, but show light metachromasia with the methylene blue counterstain. Bar = 100 μm. Figure1d, Serial section of UIP/IPF lung in Figure1b treated for the immunohistochemical localization of Wnt7B after being exposed for 30 minutes to hyaluronidase digestion. The same fibroblastic foci (FF) have lost the strong, extracellular reactivity seen in Figure1b, while there is enhanced cellular reactivity throughout the tissue, presumptively due to unmasking of intracellular, glycosylated Wnt7B. Methylene blue counterstain. Bar = 100 μm. Figure1e-f, Serial sections of UIP/IPF lung treated for the immunohistochemical localization of Wnt7B; the first (e) without and second (f) with the antigenic blocking peptide simultaneously with the Wnt7B-specific antibody. The two positive fibroblastic foci (FF) in (e) show diminished selective staining for Wnt7B in (f). Methylene blue counterstain. Bar = 100 μm.

Lungs with normal pulmonary architecture (group 1) had weak to moderate Wnt7B immunoreactivity in the cytoplasm of airway epithelium (ciliated cells) and smooth muscle of airways and arteries (Figure1a and inset). Much weaker Wnt7B reactivity was evident in the cytoplasm of AT2 cells (Figure1a inset), alveolar macrophages, and endothelial cells. Lungs originally categorized with ILD and UIP/IPF (by the LTRC) had intense and discrete immunoreactive sites for Wnt7B that varied in size and location. The largest sites correlated directly with easily identifiable fibroblastic foci characteristic of IPF, where staining was particularly intense in the extracellular matrix (Figures 1b1e2b2c2e). All sites, regardless of size and location, were unreactive with normal goat serum controls (Figure1c). Pretreatment of sections with 5% hyaluronidase prior to immunostaining for Wnt7B completely attenuated the intense extracellular matrix reactive sites seen in parallel sections, while most cellular reactivity was retained and somewhat intensified (Figure1d). This was particularly true of smooth muscle cells (data not shown). This increased cellular staining is likely due to the antibody detection of intracellular and cell surface-associated forms of the antigen, which is glycosylated [25], and made more available by the digestion procedure. 

**Figure 2:**
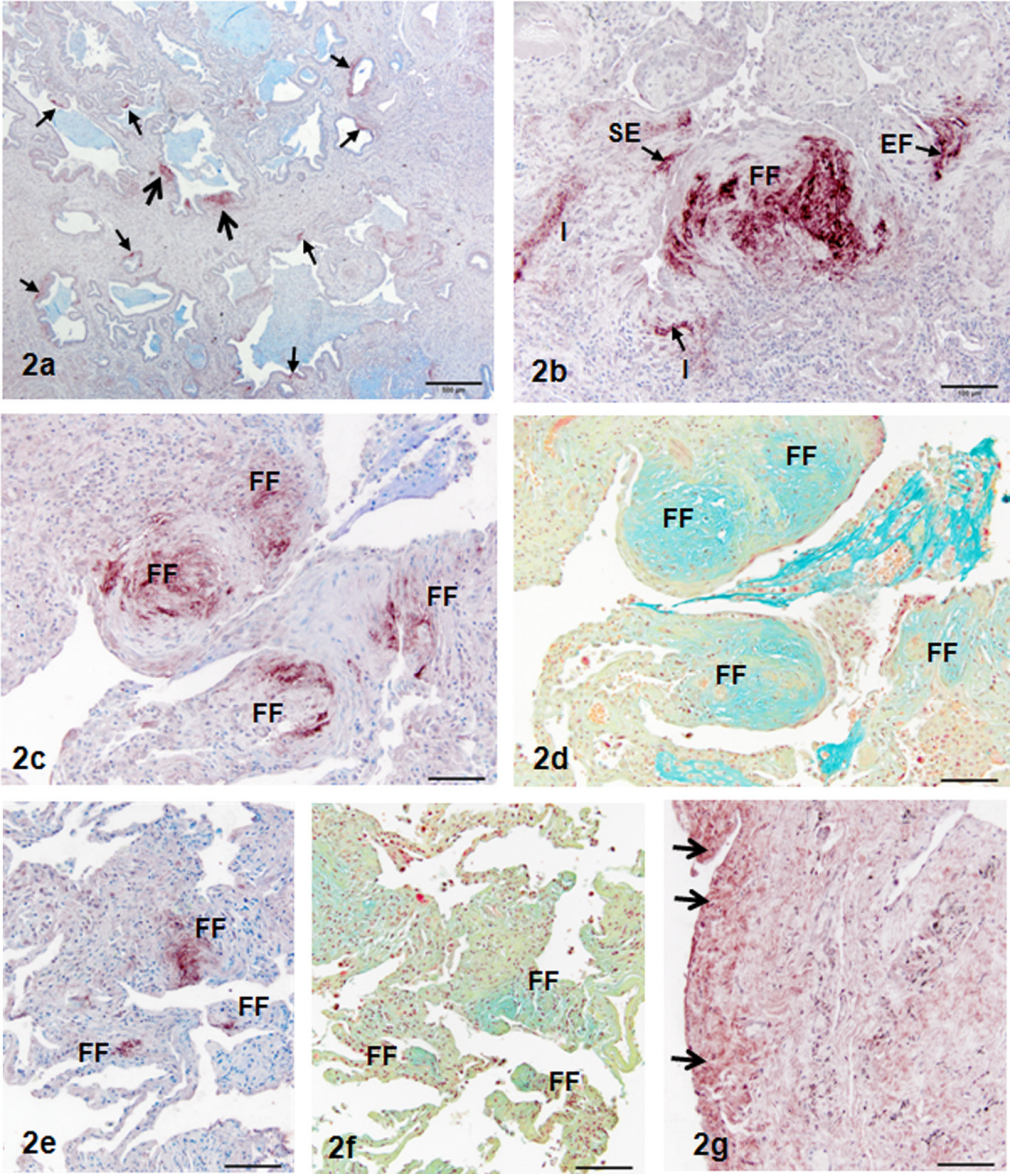
**Low power magnification of a section of UIP/IPF lung treated for the immunohistochemical localization of Wnt7B.** Fibroblastic foci are easy to identify (large arrows), as are even small reactive sites (small arrows). Methylene blue counterstain. Bar = 600 μm. Figure2b, Section of UIP/IPF lung treated for the immunohistochemical localization of Wnt7B demonstrating the three categories designated for sizing and quantitation. Preliminary histopathologic evaluation of the slides determined that all reactive sites were confined to one of three characteristic types: epithelial + fibroblast + extracellular matrix (EF), subepithelial (SE, fibroblast + extracellular matrix), or interstitial (I, not adjacent to epithelial surfaces). Methylene blue counterstain. Bar = 100 μm. Figure2c-f, Serial sections of UIP/IPF lung treated for the immunohistochemical localization of Wnt7B (c, e) or Movat’s pentachrome (d, f) in airway (c, d) and alveolar (e. f) regions. The same fibroblastic foci (FF) stain clearly with both treatments. Methylene blue counterstain. Bar = 100 μm. Figure2g, Section of UIP/IPF lung treated for the immunohistochemical localization of Wnt7B demonstrating reactivity of the mesothelial surface (arrows) and the subjacent cells and thickened interstitium. Methylene blue counterstain. Bar = 100 μm.

Simultaneous treatment of sections with Wnt7B antibody and its competitive binding Wnt7B peptide during the staining procedure resulted in significant reduction in all reactive sites in parallel sections, particularly in sites with the most intense immunoreactivity (Figure1e vs. 1f), confirming the specificity of the staining.

Even at lower magnifications, the Wnt7B reactive sites were readily detectable, especially the fibroblastic foci (Figure2a). Reactive sites were consistently found in one of three regional dispositions according to the criteria described above: EF, SE (which included most fibroblastic foci), and I (Figure2b). Sequential and non-sequential serial sections were used to compare Wnt7B stained slides with those stained with H&E, picrosirius red (PSR), Movat’s pentachrome, or immunohistochemistry for smooth muscle actin. Fibroblastic foci were easily detected by their large size (typically >100 μm^2)^ and strong extracellular reactivity for Wnt7B, with most found adjacent to thickened airways (Figure2b-c) and thickened interstitium in alveolar regions (1e, 2e). They correlated well with sequential/non-sequential serial sections stained with H&E (data not shown) and pentachrome (2d, 2f). The red reactivity of collagen with PSR was very strong throughout IPF lungs, especially in highly thickened areas of mature collagen and thickened basal laminae (data not shown). Fibroblastic foci were easily identifiable, mainly by virtue of their lighter staining, immature collagen content (as shown with the Movat’s pentachrome), which gave it a finely layered appearance (data not shown).

**Figure 3:**
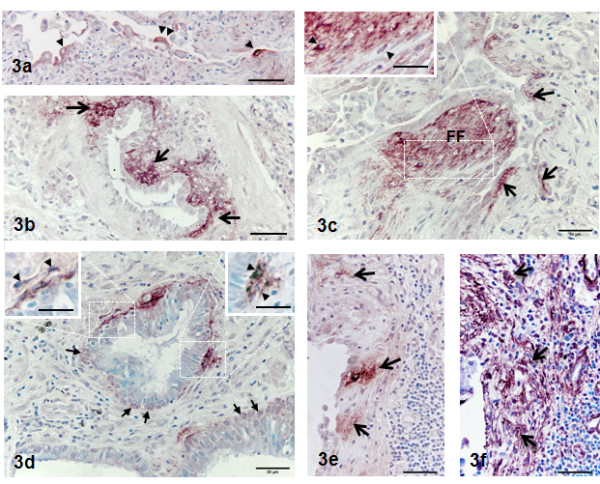
**Sections of UIP/IPF lung treated for the immunohistochemical localization of Wnt7B.** Figure3a demonstrates staining of hypertrophic/metaplastic alveolar epithelial cells. Figure3b demonstrates extensive subepithelial (SE, not an FF) site reactivity (arrows) around one side of a small airway. Figure3c demonstrates reactivity within a fibroblastic focus (FF) and adjacent SE sites (arrows). The inset at higher magnification of the fibroblastic foci demonstrates the extracellular matrix and cellular (arrow) nature of the reactivity. Figure3d demonstrates extensive subepithelial (SE) site reactivity (arrows) around two small airways, the apparent reactivity of presumptive basal cells (arrows) within the respective airways. The inset at higher magnification shows presumptive, reactive fibroblasts embedded within reactive matrix (arrowheads). Methylene blue counterstain. Bars = 50 μm; inset bars = 30 μm.

Wnt7B immunoreactivity was found both intra- and extracellularly. Without hyaluronidase digestion, the extracellular reactivity was discrete and intense and found in the ECM regions of fibroblastic foci (Figures 1b, 1e, 2b-c, 2f), subepithelial ECM (Figure2a-b, 3b), and interstitium (Figure2b) not adjacent to epithelium. The sizes of the reactive sites varied widely, with fibroblastic foci having the greatest individual reactive areas (often >1600 μm^2^; Figures 1b, 2c, 2b-c, 2e) and the SE regions being the most numerous and smallest in individual reactive area (Figure2b, 3a-d). These smaller sites were not distinguishable by any morphologic features or special staining characteristics (data not shown). Intracellular reactivity was very intense in some epithelia, especially that considered hyper- or metaplastic (Figure3a), and in fibroblasts of fibroblastic foci, especially the larger SE sites (Figure3c) and smaller SE sites (Figure3d). In airways, basal cells were often reactive (Figure3d), as were some ciliated cells (data not shown). Mesothelium was generally positive, but mesothelial immunoreactivity was most intense when overlying areas of interstitial fibrosis (Figure2g). Macrophages were variably reactive for Wnt7B, while other inflammatory cells present in IPF/UIP lungs were uniformly negative (data not shown). Many SE sites reflected staining of portions of basal lamina (Figures 3b-d) and/or strong staining of fibroblasts within the subepithelial fibroblast layer (Figure3d). The fibroblastic component of Wnt7B-positive fibroblastic foci (Figure3d-e) was uniformly positive for smooth muscle actin (Figure3f), reflective of the myofibroblast phenotype.

**Figure 4:**
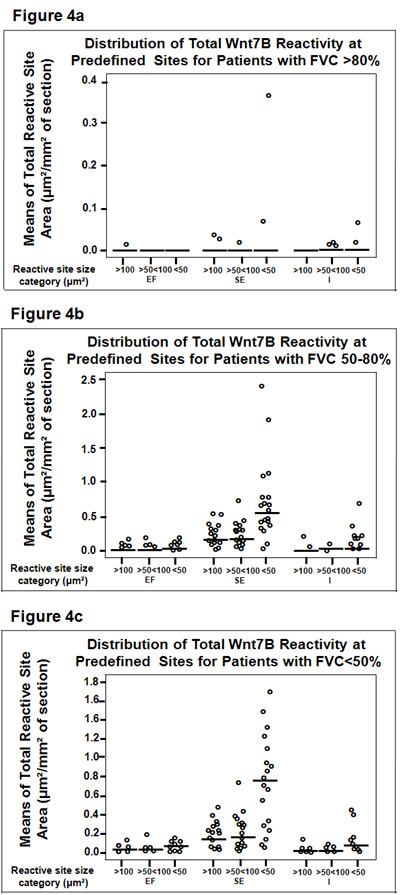
**Distribution of the total Wnt7B reactivity at the EF, SE, and I site categories for patients with FVCs >80% (normal), 50-80% (intermediate impairment), or <50% (severe impairment).** Normal patients had minimal Wnt7B reactivity, most with a median total area reactivity of “0” (a). Patients with impaired FVCs had many more reactive sites than controls, although those with intermediate and severe impairment were not different (see Tables 2 and 3). Notably, the median of the mean total of reactivity was greatest in the SE sites that were <50 μm² (b-c).

Patients with FVC > 80% had few quantifiable Wnt7B reactive sites (Figure4a) compared to patients with functional diagnoses of IPF/UIP [<80% (Figures 4b-c)]. There were insufficient reactive sites for the EF category to compare to corresponding reactive SE and I regions. Despite an outlier (defined as greater than 2 standard deviations from the mean) for the SE region <50 μm^2^ category, the reactive sites for locations SE and I were not statistically different, given that the median value was 0 for all reactive sites by location. For patients with FVC 50-80% and FVC <50%, reactive site size means (in μm^2^), standard deviations, and minimum, median, and maximum sizes of reactive sites (in μm^2^) are presented in Tables 2 and 3. For patients with FVC 50-80% and FVC <50%, the means of the number of each of the reactive site sizes for the SE location were statistically greater than that of those for EF and I locations (p < 0.05).

**Table 2:** Patients with FVC > 50% < 80%

**Site/size**	**Mean**	**StDev**	**Min**	**Med**	**Max**
EF > 100	0.02098	0.03446	0	0.00906	0.14388
EF >50 < 100	0.01925	0.03713	0	0.00829	0.17004
EF <50	0.0427	0.0477	0	0.0181	0.1653
**SE >100**	*0.198	**0.1508**	**0**	**0.1613**	**0.5167**
**SE >50 < 100**	*0.194	**0.1715**	**0**	**0.1575**	**0.7086**
**SE <50**	*0.675	**0.562**	**0**	**0.537**	**2.38**
I >100	0.02319	0.04121	0	0.00829	0.186
I >50 < 100	0.0231	0.02473	0	0.01027	0.08267
I <50	0.1044	0.152	0	0.0458	0.6613

**Table 3:** Patients with FVC < 50%

**Site/size**	**Mean**	**StDev**	**Min**	**Med**	**Max**
EF >100	0.02615	0.02849	0	0.0159	0.10568
EF >50 < 100	1.02039	0.02286	0	0.01761	0.07789
EF <50	0.04478	0.03933	0	0.04559	0.124
**SE >100**	*0.1589	**0.03933**	**0.00677**	**0.131**	**0.4524**
**SE >50 < 100**	*0.2121	**0.167**	**0.0124**	**0.1976**	**0.6228**
**SE <50**	*0.7170	**0.477**	**0.0352**	**0.755**	**1.654**
I >100	0.01564	0.02594	0	0.0124	0.11683
I >50 < 100	0.02883	0.03709	0	0.02067	0.15578
I <50	0.093	0.1135	0	0.0671	0.4284

* the means of the number of each of the reactive site sizes for the SE location were statistically greater than that of those for EF and I locations (p < 0.05).

## Discussion

The initiating cause(s) underlying the pathogenesis of idiopathic pulmonary fibrosis (IPF) is unknown. Much attention has focused on a failed repair of alveolar epithelium, and its impact on subsequent loss of pulmonary architecture. This failure manifests as the inability of epithelial surfaces to proliferate and differentiate effectively resulting in hyperplasia, metaplasia, and/or transdifferentiation into myofibroblast-like cells [26]. Exactly how these dysplastic events culminate, independently or collectively, in the formation of one of the hallmarks of IPF, the fibroblastic focus, is not clear. Studying the roles that specific signaling pathways play in the complex processes involved in the formation of fibroblastic foci has led to a greater understanding of important growth factors, such as TGF-β, that not only promote the deposition of excessive collagen but mediate the process of EMT as well [10]. TGF-β has been immunohistochemically localized to alveolar epithelium and extracellular matrix of IPF lungs [27] and is within fibroblastic foci [28,29]. Fibroblastic foci are also rich in its downstream nuclear targets like phospho-Smad 2/3 [28] and relevant proteins, such as cysteine-rich protein 1 (CRP-1), which is important in smooth muscle cell differentiation [30]. Relatedly, activated β-catenin has been shown to be up-regulated in the nuclei of myofibroblasts of fibroblastic foci of IPF patients [14], and alveolar epithelial responses to TGF-β involve alpha3 integrin for β-catenin phosphorylation and formation of a β-catenin/p-Smad2 complex resulting in initiation of EMT [31]. More specifically, TGF-β-stimulated Smad3 has recently been shown to form a complex with β-catenin and CREB-binding protein in regulation of α-smooth muscle actin, a cytologic signature of EMT [32]. SPARC (secreted protein acidic rich in cysteine), an extracellular matrix component abundant in fibroblastic foci [33], should also be noted, as it has been shown to activate AKT, inhibit GSK-3β, and activate β-catenin, resulting in an anti-apoptotic phenotype [34], another characteristic of the IPF myofibroblast. [10-12].

The finding of strong Wnt7B immunoreactivity in the fibroblastic focus of UIP/IPF lungs would seem to fit readily with the above discussion on canonical Wnt signaling involving β-catenin. The demonstration of Wnt7B in dysplastic airway and alveolar epithelium and in myofibroblasts of the fibroblastic foci closely correlates with the site localization of active β-catenin in IPF lungs [14]. However, that study did not report active β-catenin in fibroblasts/myofibroblasts in regions other than fibroblastic foci. Wnt7B localization, demonstrated here, was also clearly defined in a large number of smaller sites (<50 μm^2^) that, like fibroblastic foci, had both cellular and extracellular matrix components. The loss of extracellular Wnt7B immunoreactivity after hyaluronidase digestion supports the matrix-associated localization, which is not surprising, as Reichsman, et al., [24] have demonstrated that most secreted Wnts (approximately 83%) are bound to the cell surface and surrounding extracellular matrix through specific, non-covalent interactions. The localization of Wnt7B in fibroblastic foci and a large number of smaller subepithelial sites position it well for influencing the activity of the underlying interstitium. In normal lung development, murine Wnt7b has been shown to be exclusively expressed in epithelial cells and regulated by TTF-1, GATA-6, and FoxA2 [35]. In the embryo, it has been demonstrated to stimulate epithelial and mesenchymal proliferation, likely through its activation of *BMP-4* and *Id2*[36]. Further, Wnt7b/β-catenin signaling regulates a program of mesenchymal cell differentiation and proliferation that is necessary for smooth muscle cell development in cooperation with tenascin-C and involving PDGFR-alpha and PDGFR-beta [37]. It may not be surprising, then, that similar genetic programs are reactivated in some form in an adult disease such as IPF, in which tenascin-C is heavily expressed in the matrix of fibroblastic foci [33] where myofibroblasts expressing PDGFRs are located [38]. Coupled with the strong expression of Wnt7B in hyperplastic/metaplastic epithelium and fibroblastic foci reported here, these observations support previous conclusions that epithelium in IPF may be responsible for aberrant activation of Wnt signaling, such as that of Wnt7B, in adjacent mesenchyme, leading to damage to the lung and fibrosis [19].

The notion that Wnt7B may have a potentially contributive if not significant role in the development and/or progression of IPF draws on previous assumptions that the fibroblastic foci are central to the disease process and its prognosis [39,40]. The fibroblastic foci were the most dramatic sites of Wnt7B localization observed in the IPF lung specimens. However, when systematically categorized and analyzed relative to location and size, the Wnt7B reactive sites of large fibroblastic foci were not the most numerous nor did they consume the greatest percentage of total tissue section area, regardless of disease severity according to % FVC, assuming the section samples correctly reflected the overall extent of fibrotic change of the whole lung (see Figure4). This distinction belonged to the small subepithelial (SE) sites <50 μm^2^ (Figures 1d2a3b-d). These unique reactive sites had no other morphological features that were specifically distinguishing other than their discrete Wnt7B immunoreactivity. In parallel sections, the small SE sites, like the larger fibroblastic foci, consistently contained abundant cells expressing the myofibroblast phenotype, as indicated by α-smooth muscle actin immunoreactivity (Figure3f), but by virtue of their Wnt7B reactivity, clearly represented a subpopulation of these cells compared with the overall total myofibroblast population in IPF lungs. While it is not clear from the current data that there are any connections between the Wnt7B positive reactivity of the fibroblastic foci and the more numerous, smaller SE sites, Cool, et al., [41] demonstrated with three-dimensional reconstruction of pentachrome-stained sections that fibroblastic foci of UIP form a complex, interconnected network that extends from the pleura into the parenchyma. Perhaps the strong reactivity for Wnt7B within fibroblastic foci, shown here to correlate precisely with pentachrome reactivity, along with the foci’s complement of established pro-fibrogenic components constitute the expansion unit of IPF, and the small Wnt7B-positive, subepithelial (SE) sites are the leading edge of this process. The location of the small SE sites could portend the epithelial-fibroblast cross-talk often involving Wnt signaling and known to be important determinants of the fibrogenic events characteristic of IPF [26]. The regionally confined localization of Wnt7B differs from that of Wnt5A, which is strongly expressed in the majority of fibroblasts derived from UIP patients (i.e., almost all of the remaining fibroblasts), and shown to signal through non-canonical pathways, promote proliferation, and prevent apoptosis [42]. It raises the possibility that Wnt7B and Wnt5A, along with TGF-β, SPARC, and tenascin-C, work in some coordinated or concerted fashion to modulate fibroblast/myofibroblast activities in adjacent and/or different anatomic regions of IPF lungs. Extensive and more detailed studies are currently underway to help develop a better understanding of this complex process.

## Conclusions

These observations draw attention to a specific Wnt signaling ligand, Wnt7B, which by virtue of its established roles in epithelial and mesenchymal proliferation and differentiation, procollagen production, and enhanced gene expression in IPF, would be expected to act as a significant contributor to the pathogenesis of IPF. This is supported not only by the strong expression of Wnt7B in fibroblastic foci but also by the numerous, small subepithelial sites that may represent early stages of developing fibroblastic foci as part of a larger, expanding network of fibrogenic tissue.

## Competing interests

There are no competing interests to report by any of the authors.

## Authors’ contributions

TM – performed immunostaining, prepared photographic images, drafting and revision of manuscript; AH - performed immunostaining, KF - developed protocol staining and performed immunostaining; BG - performed immunostaining; JT – developed immunostainig protocol; DN - interpretation of data, drafting and revision of manuscript; SJ – analysis and interpretation of data, manuscript revision; MC - analysis and interpretation of data, statistical analysis; PS – conception and design of study, analysis and interpretation of data, preparation of photographic images, drafting of manuscript and revision; all authors read and approved of the final manuscript.

## Aknowledgments

This investigation was supported by PHS grants HL44497, HL95411, North Carolina State University College of Veterinary Medicine, and the State of North Carolina. The authors wish to thank Thomas Sporn, M.D., Department of Pathology, Duke University Medical Center, Durham, NC, for his guidance and diagnostic support, and Laura Shewmon, Department of Population Health and Pathology, College of Veterinary Medicine, North Carolina State University for her technical contribution to this study.
